# Dehydroepiandrosterone Supplementation Improves the Outcomes of *in vitro* Fertilization Cycles in Older Patients With Diminished Ovarian Reserve

**DOI:** 10.3389/fendo.2019.00800

**Published:** 2019-11-15

**Authors:** San-Nung Chen, Kuan-Hao Tsui, Peng-Hui Wang, Chyi-Uei Chern, Zhi-Hong Wen, Li-Te Lin

**Affiliations:** ^1^Department of Obstetrics and Gynecology, Kaohsiung Veterans General Hospital, Kaohsiung City, Taiwan; ^2^Department of Obstetrics and Gynecology, National Yang-Ming University School of Medicine, Taipei City, Taiwan; ^3^Department of Pharmacy and Master Program, College of Pharmacy and Health Care, Yanpu Township, Taiwan; ^4^Department of Obstetrics and Gynecology, Taipei Veterans General Hospital, Taipei City, Taiwan; ^5^Department of Medical Research, China Medical University Hospital, Taichung City, Taiwan; ^6^Department of Marine Biotechnology and Resources, National Sun Yat-sen University, Kaohsiung City, Taiwan; ^7^Department of Biological Science, National Sun Yat-sen University, Kaohsiung City, Taiwan

**Keywords:** dehydroepiandrosterone, DHEA, diminished ovarian reserve, *in vitro* fertilization, poor ovarian responders, POSEIDON group

## Abstract

**Background:** Dehydroepiandrosterone (DHEA) supplementation has been reported to have beneficial effects on the *in vitro* fertilization (IVF) outcomes of patients with poor ovarian response or diminished ovarian reserve. The Patient-Oriented Strategies Encompassing IndividualizeD Oocyte Number (POSEIDON) stratification is a set of newly established criteria for low prognosis patients. The aim of this study was to examine the potential effects of DHEA supplementation on the IVF outcomes of patients who fulfill the POSEIDON group 4 criteria.

**Methods:** This retrospective cohort study investigated 297 cycles that fulfilled the POSEIDON group 4 criteria and underwent IVF treatment using the gonadotropin-releasing hormone antagonist protocol. The study group contained 159 cycles that received DHEA (30 mg three times per day) daily for 12 weeks before their IVF cycles. The control group included 138 cycles that underwent IVF cycles but did not receive DHEA. The baseline characteristics and cycle parameters as well as the IVF outcomes of both groups were compared.

**Results:** In terms of baseline characteristics, more previous IVF attempts and lower AMH levels were found in the study group than in the control group. Regarding IVF outcomes, patients in the study group had significantly higher follicular oocyte index and higher numbers of retrieved oocytes, metaphase II oocytes, fertilized oocytes, day 3 embryos and top-quality day 3 embryos than those in the control group. Besides, a higher cumulative pregnancy rate and lower cancellation rate were observed in the study group than in the control group although clinical pregnancy rate, live birth rate, and cumulative live birth rate did not differ between the two groups. Whether patients are aged ≤ 40 years or aged > 40, higher numbers of oocytes and embryos were observed in the study group than in the control group. In patients aged > 40, cumulative pregnancy rate was significantly higher in the study group than in the control group.

**Conclusions:** Our data suggest that DHEA supplementation might increase both oocyte and embryo yields, as well as cumulative pregnancy rates, in patients fulfilling the POSEIDON group 4 criteria.

## Introduction

Dehydroepiandrosterone (DHEA) is an endogenous steroid chiefly originating from the adrenal zona reticularis and ovarian theca cells in women (1). DHEA acts as an essential prohormone in the production of testosterone (1), which is believed to be involved in early folliculogenesis (2). Since Casson et al. (3) first reported that DHEA supplementation in poor ovarian responder (POR) contributes to an enhanced ovarian response, numerous studies (4–9) and meta-analyses (10–12) have shown its beneficial effects in POR, leading to an improved ovarian reserve, an increased oocyte yield and quality, and a higher pregnancy rate or live birth rate following *in vitro* fertilization (IVF) treatment. However, these studies used different definitions of POR, so the results were not clear evidence of DHEA effectiveness.

Indeed, large discrepancies in the definition of POR exist in previous studies (13). In 2011, the ESHRE consensus (14) released the Bologna criteria to standardize the definition of POR, defining POR when at least two of the following three features are present: (i) advanced maternal age (≥40 years) or any other risk factor for POR; (ii) a previous poor ovarian response (≤3 oocytes with a conventional stimulation protocol); and (iii) an abnormal ovarian reserve test (antral follicle count [AFC] <5–7 follicles or anti-Müllerian hormone [AMH] <0.5–1.1 ng/ml). Additionally, two episodes of a previous POR after maximal stimulation alone would be sufficient to classify a patient as having POR. However, the Bologna criteria have been criticized for several limitations. Heterogeneity of the population is the main drawback of the Bologna criteria, which comprise various subpopulations with diverse baseline characteristics (15). Moreover, the specific risk factors for POR are not clearly defined. The threshold values of AFC and AMH are not standardized and have clear cut-off levels (16).

In 2016, the Patient-Oriented Strategies Encompassing IndividualizeD Oocyte Number (POSEIDON) group proposed a new stratification, suggesting a new concept of low prognosis to promote a personalized approach (17). However, few studies have investigated the effects of DHEA based on the POSEIDON stratification in IVF cycles. POSEIDON group 4 refers to the group of older patients with diminished ovarian reserve. This study attempts to explore the potential effects of DHEA treatment on the IVF outcomes of patients who fulfill the POSEIDON group 4 criteria.

## Materials and Methods

### Study Design and Participants

**Figure 1:**
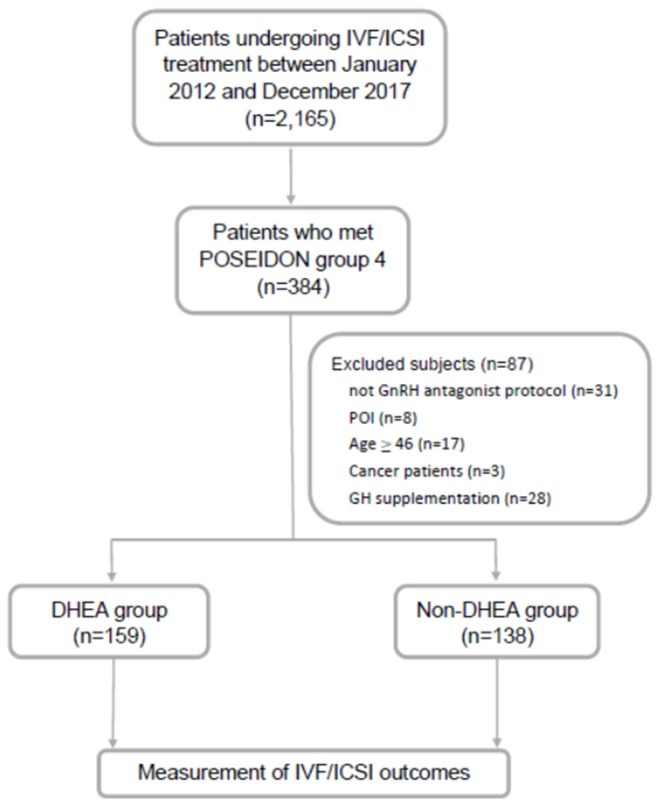
Flow chart of the study design. IVF, *in vitro* fertilization; ICSI, intracytoplasmic sperm injection; GnRH, gonadotropin-releasing hormone; GH, growth hormone; POI, primary ovarian insufficiency; DHEA, dehydroepiandrosterone.

This retrospective cohort study was conducted at the Reproductive Center of the Kaohsiung Veterans General Hospital from January 2012 to December 2017. Patients who underwent IVF cycles and fulfilled the POSEIDON group 4 criteria were included in this study. The POSEIDON group 4 criteria were age ≥ 35 years and AFC < 5 or AMH <1.2 ng/ml. The exclusion criteria were as follows: (i) patients who did not undergo gonadotropin-releasing hormone (GnRH) antagonist protocol, (ii) patients who were diagnosed with primary ovarian insufficiency, (iii) patients with an extremely advanced age (age ≥ 46 years), (iv) patients who suffered from malignancy, and (v) patients who received growth hormone supplementation. Finally, 297 IVF cycles were identified and allocated to either the DHEA (*n* = 159) or non-DHEA (*n* = 138) group. In the DHEA group, DHEA 30 mg three times a day (CPH; Formulation Technology, Oakdale, CA, USA) was administered for 3 months prior to the subsequent IVF cycle. In the non-DHEA group, patients commenced an IVF cycle without pretreatment with DHEA. The choice of DHEA supplementation depended on the patient's consideration and preference after a full consultation provided by a doctor. The study flow chart is shown in Figure 1.

### Treatment Protocol

Only patients undergoing the GnRH antagonist protocol were included in this study. Within 5 days of a spontaneous menstrual cycle, an ultrasound was performed to determine the AFC. Gonadotropins, including recombinant follicle stimulating hormone (rFSH, Gonal-F, Merck Serono S.p.A., Modugno, Italy), rFSH plus recombinant luteinizing hormone (Pergoveris, Merck Serono SA, Aubonne, Switzerland), human menopausal gonadotropin (Merional, IBSA Institut Biochimique S.A., Lamone, Switzerland), or corifollitropin alfa (Elonva, Vetter Pharma-Fertigung GmbH & Co, KG, Ravensburg, Germany) were started for controlled ovarian stimulation if suitable.

GnRH antagonists (Cetrotide 0.25 mg, Pierre Fabre Medicament Production, Aquitaine Pharm International, Idron, France or Orgalutran 0.25 mg, Vetter Pharma-Fertigung GmbH & Co, KG, Ravensburg, Germany) were initiated when the leading follicle reached a diameter of 12–14 mm. When the leading follicle was 18 mm or greater, final oocyte maturation was triggered by either recombinant human chorionic gonadotropin (hCG, Ovidrel 250 μg, Merck Serono S.p.A., Modugno, Italy) alone or with a dual trigger comprising recombinant hCG and GnRH agonist (Lupro 2 mg, Nang Kuang Pharmaceutical Co, Ltd., Tainan, Taiwan). The choice of hCG alone or the dual trigger depended on the physician's preference. Oocyte aspiration was conducted by transvaginal ultrasound guidance 34–36 h after the oocyte trigger. Whether fertilization was conducted by conventional IVF or intracytoplasmic sperm injection depended on a semen analysis or prior failure of fertilization. Embryos were monitored for cell number and morphological quality based on the Istanbul consensus workshop criteria (18). All embryos were frozen by vitrification on the third day after oocyte retrieval for subsequent transfer.

Regarding endometrial preparation for cryopreserved embryo transfer, hormone replacement therapy with oral estradiol (Ediol 8 mg, Synmosa Biopharma Corporation, Hsinchu County, Taiwan) plus estradiol gel (Oestrogel gel, Besins, Drogenbos, Belgium) per day was initiated within cycle day 5. When the endometrial thickness reached at least 8 mm, daily progesterone, including intravaginal gel (Crinone 8% gel, Merck Serono, Hertfordshire, UK) and oral dydrogesterone (Duphaston 40 mg, Abbott, Olst, the Netherlands), were given simultaneously. Then, embryo transfer was scheduled under the guidance of transabdominal sonography. Once pregnancy was confirmed, progesterone was continued until 8–10 weeks of gestation.

### Outcome Measures

The primary outcomes of this study were the numbers of retrieved oocytes and mature oocytes. The secondary outcomes included the numbers of fertilized oocytes, day 3 embryos, and top-quality day 3 embryos and clinical pregnancy rate, cumulative pregnancy rate, live birth rate, and cumulative live birth. Clinical pregnancy was determined by the visualization of the intrauterine sac with a fetal heartbeat by transvaginal ultrasound. Cumulative pregnancy was defined as at least one visible fetal heartbeat by transvaginal ultrasound resulting from one initiated or aspirated IVF cycle including all subsequent frozen/thawed IVF cycles. Live birth was determined as an infant born alive after 20 weeks of gestation. Cumulative live birth was defined as at least one live born baby resulting from one initiated or aspirated IVF cycle including all subsequent frozen/thawed IVF cycles.

### Statistical Analysis

The Statistical Package for Social Sciences (SPSS) version 20.0 (Chicago, IL, USA) was applied for all analyses. Numerical data were assessed for normality with the Kolmogorov-Smirnov test. Continuous measurements are presented as the mean ± standard deviation; qualitative measurements are presented as percentages. Continuous data were compared by means of Student's *t*-test; categorical data were compared using chi-squared tests. Moreover, binary logistic regression was used to evaluate the odds ratios (ORs) and 95% confidence intervals (CIs) when the number of retrieved oocytes was more than 3 after adjusting for confounders. Statistical significance was defined as *p* < 0.05.

## Results

As shown in Figure 1, out of 2,165 IVF cycles, 384 fulfilled the POSEIDON group 4 criteria (17.7%). After excluding 87 cycles, including those of patients not receiving the GnRH antagonist protocol (*n* = 31), with primary ovarian insufficiency (*n* = 8), aged ≥ 46 (*n* = 17), with cancer (*n* = 3) and with growth hormone supplementation (*n* = 28), the remaining 297 cycles were identified and then divided into two groups based on whether or not there was DHEA supplementation: the DHEA (*n* = 159) and non-DHEA (*n* = 138) groups.

Table 1 summarizes the baseline characteristics of the included cycles. The age, body mass index, infertility duration, types of infertility, basal FSH levels and AFCs were similar without any statistically significant differences between the DHEA and non-DHEA groups. Nevertheless, more previous IVF attempts and lower AMH levels were observed in the DHEA group than in the non-DHEA group.

**Table 1:** Baseline characteristics of the older patients with diminished ovarian reserve (POSEIDIN group 4) treated with or without DHEA.

**Parameters**	**DHEA**	**Non-DHEA**	***p*-value**
	**(*n* = 159)**	**(*n* = 138)**	
Age (years)	39.8 ± 2.5	40.2 ± 2.9	0.295
Body mass index (kg/m^2^)	22.3 ± 3.1	23.0 ± 3.7	0.054
Infertility duration (years)	5.6 ± 4.6	4.9 ± 4.3	0.177
Previous IVF attempts (n)	2.5 ± 2.7	1.3 ± 1.6	< 0.001
Types of infertility (%)			0.166
Primary infertility	43.4	51.4	
Secondary infertility	56.6	48.6	
Basal FSH (IU/l)	7.3 ± 4.5	6.3 ± 4.1	0.082
Antral follicle counts (n)	4.5 ± 2.4	4.2 ± 2.3	0.342
Anti-Müllerian hormone (ng/ml)	0.5 ± 0.3	0.7 ± 0.3	0.003

Data are presented as mean ± standard deviation or %.

*DHEA, dehydroepiandrosterone; IVF, in vitro fertilization; FSH, follicle stimulation hormone*.

As presented in Table 2, there were no significant differences regarding the stimulation duration, types of gonadotropins, percentage of combination with corifollitropin alfa, gonadotropin dose, or method of oocyte triggering. Compared to those in the control group, the patients receiving DHEA supplementation showed significant improvements in several parameters, including follicular oocyte index (58.8 ± 36.9% vs. 43.5 ± 35.2%, *p* < 0.001), the numbers of retrieved oocytes (3.1 ± 2.8 vs. 1.9 ± 1.8, *p* < 0.001), metaphase II oocytes (2.6 ± 2.1 vs. 1.7 ± 1.5, *p* < 0.001), fertilized oocytes (2.1 ± 2.0 vs. 1.4 ± 1.5, *p* = 0.001), day 3 embryos (1.9 ± 1.9 vs. 1.3 ± 1.4, *p* = 0.001) and top-quality day 3 embryos (0.8 ± 1.3 vs. 0.4 ± 0.7, *p* = 0.001). Patients in the DHEA group had higher percentage of blastocyst transfer than those in the non-DHEA group (13.8% vs. 5.1%, *p* = 0.011). Furthermore, the number of frozen embryo transfer cycles (1.1 ± 0.9 vs. 0.8 ± 0.6, *p* < 0.001) and percentage of cycles in which more than one embryo was available for transfer (36.3% vs. 10.9%, *p* < 0.001) were significantly higher in the DHEA group than in the non-DHEA group. Although the two groups were similar in terms of the day 3 embryos rate, clinical pregnancy rate, live birth rate and cumulative live birth rate, a higher cumulative pregnancy rate (29.5% vs. 14.8%, *p* = 0.011) and lower cancellation rate (18.9% vs. 29.7%, *p* = 0.029) were observed in the DHEA group than in the non-DHEA group.

**Table 2:** Cycle characteristics and pregnancy outcomes of older patients with diminished ovarian reserve (POSEIDIN group 4) treated with or without DHEA.

**Parameters**	**DHEA**	**Non-DHEA**	***p*-value**
	**(*n* = 159)**	**(*n* = 138)**	
Stimulation duration (days)	10.7 ± 2.3	10.3 ± 2.4	0.139
Types of gonadotropins			0.350
rFSH only	6.9%	7.2%	
rFSH/rLH	61.6%	68.8%	
HMG	31.4%	23.9%	
Combination with corifollitropin alfa (%)	21.4%	21.7%	0.941
Gonadotropin dosage (IU)			
With corifollitropin alfa	1727.6 ± 515.9	1440.0 ± 997.5	0.051
Without corifollitropin alfa	2839.0 ± 834.6	2929.3 ± 834.6	0.456
Oocyte trigger (%)			0.102
Dual trigger	81.1	73.2	
hCG trigger	18.9	26.8	
No. of retrieved oocytes (*n*)	3.1 ± 2.8	1.9 ± 1.8	<0.001
Follicular oocyte index (%)	58.8 ± 36.9	43.5 ± 35.2	<0.001
No. of retrieved oocytes > 3 (%)	33.3	19.6	0.008
No. of metaphase II oocytes (*n*)	2.6 ± 2.1	1.7 ± 1.5	<0.001
No. of fertilized oocytes (*n*)	2.1 ± 2.0	1.4 ± 1.5	0.001
No. of Day 3 embryos (*n*)	1.9 ± 1.9	1.3 ± 1.4	0.001
Day 3 embryos rate (%)	94.0 ± 18.0	94.2 ± 17.2	0.935
No. of top-quality Day 3 embryos (*n*)	0.8 ± 1.3	0.4 ± 0.7	0.001
Day of embryo transfer			0.011
Day 3	86.2%	94.9%	
Day 5	13.8%	5.1%	
No. of embryo transfer (*n*)	1.1 ± 0.9	0.8 ± 0.6	<0.001
No. of embryo transfer > 1 (%)^*^	36.3%	10.9%	<0.001
Clinical pregnancy rate (%) / per cycle	17.6	11.6	0.146
Cumulative pregnancy rate (%)	29.5	14.8	0.011
Live birth rate (%) / per cycle	11.9	9.4	0.483
Cumulative live birth rate (%)	20.0	12.0	0.120
Cancellation rate (%)	18.9%	29.7%	0.029

Data are presented as mean ± standard deviation or %.

DHEA, dehydroepiandrosterone; rFSH, recombinant follicle stimulating hormone; rLH, recombinant luteinizing hormone; HMG, human menopausal gonadotropins; hCG, human chorionic gonadotrophin.

* *only transferable cycles were calculated*.

As presented in Table 3, a binary logistic regression was performed to determine whether DHEA supplementation or not had a beneficial effect on retrieved oocytes more than 3. Confounding variables such as age, body mass index, infertility duration, previous IVF attempts, types of infertility, basal FSH, AFC, and AMH were included in the analysis. The multivariate analysis showed that DHEA supplementation was positively associated with retrieved oocytes more than 3 (OR = 3.37, 95% CI 1.64–6.96, *p* = 0.001). AFC (OR = 1.48, 95% CI 1.26–1.74, *p* < 0.001) and AMH level (OR = 8.78, 95% CI 2.84–27.15, *p* < 0.001) were also positively associated with the possibility of retrieving more than 3 oocytes. As shown in Table 4, regardless of whether the patients were aged ≤40 or >40 years, those receiving DHEA had higher numbers of retrieved oocytes, metaphase II oocytes, fertilized oocytes, day 3 embryos and top-quality day 3 embryos than those without DHEA. Besides, in the patients older than 40 years, cumulative pregnancy rate was significantly higher in the DHEA group than in the non-DHEA group (31.2% vs. 10.6%, *p* = 0.022).

**Table 3:** Analyses of factors affecting the retrieval of more than 3 oocytes in older patients with diminished ovarian reserve (POSEIDIN group 4).

	**Univariate analysis**		**Multivariate analysis**	
	**OR**	***p*-value**	**Adjusted OR**	***p*-value**
	**(95% CI)**		**(95% CI)**	
DHEA vs. non-DHEA	2.06 (1.21–3.51)	0.008	3.37 (1.64–6.96)	0.001
Age (years)	0.87 (0.79–0.96)	0.006	0.91 (0.80–1.04)	0.173
BMI (kg/m^2^)	0.97 (0.90–1.05)	0.505		
Infertility duration (years)	0.93 (0.87–1.00)	0.040	0.96 (0.88–1.05)	0.359
Previous IVF attempts (*n*)	0.99 (0.86–1.14)	0.874		
Types of infertility				
Primary vs. Secondary	0.98 (0.59–1.64)	0.940		
Basal FSH (IU/l)	0.97 (0.91–1.04)	0.356		
AFC (*n*)	1.57 (1.37–1.80)	<0.001	1.48 (1.26–1.74)	<0.001
AMH (ng/ml)	9.38 (3.73–23.55)	<0.001	8.78 (2.84–27.15)	<0.001

*OR, odd ratio; CI, confidence interval; DHEA, dehydroepiandrosterone; BMI, Body mass index; IVF, in vitro fertilization; FSH, follicle stimulation hormone; AFC, antral follicle counts; AMH, Anti-Müllerian hormone*.

**Table 4:** Subgroup analyses (categorized relative to the age of 40) of older patients with diminished ovarian reserve (POSEIDIN group 4) treated with or without DHEA.

	**≤40 (35~40) y/o**			**>40 (41~45) y/o**		
**Parameters**	**DHEA**	**Non-DHEA**	***p*-value**	**DHEA**	**Non-DHEA**	***p*-value**
	**(*n* = 97)**	**(*n* = 77)**		**(*n* = 62)**	**(*n* = 61)**	
Age (years)	38.3 ± 1.7	38.0 ± 1.6	0.339	42.3 ± 1.3	42.9 ± 1.3	0.017
Body mass index (kg/m^2^)	22.4 ± 3.6	23.0 ± 4.0	0.371	22.0 ± 2.0	23.1 ± 3.3	0.024
Basal FSH (IU/l)	7.3 ± 5.3	6.5 ± 4.1	0.287	7.2 ± 3.1	6.2 ± 4.1	0.131
Antral follicle counts (*n*)	4.7 ± 2.2	4.5 ± 2.3	0.532	4.2 ± 2.6	3.9 ± 2.1	0.588
Anti-Müllerian hormone (ng/ml)	0.5 ± 0.3	0.7 ± 0.3	< 0.001	0.6 ± 0.4	0.6 ± 0.3	0.629
No. of oocytes retrieved (*n*)	3.1 ± 2.5	2.1 ± 1.8	0.002	3.0 ± 3.1	1.8 ± 1.7	0.010
No. of metaphase II oocytes (*n*)	1.6 ± 1.3	1.2 ± 1.3	0.043	1.6 ± 1.8	0.9 ± 1.1	0.009
Maturation rate (%)	59.2 ± 30.9	60.2 ± 35.5	0.863	60.7 ± 32.5	54.9 ± 36.1	0.422
No. of fertilized oocytes (*n*)	2.1 ± 1.9	1.5 ± 1.5	0.019	2.0 ± 2.2	1.3 ± 1.3	0.024
Fertilization rate (%)	68.5 ± 30.4	71.5 ± 34.3	0.581	74.4 ± 30.1	78.0 ± 28.6	0.567
No. of Day 3 embryos (*n*)	2.0 ± 1.8	1.4 ± 1.5	0.011	1.8 ± 2.1	1.2 ± 1.2	0.040
No. of top-quality Day 3 embryos (*n*)	0.8 ± 1.3	0.5 ± 0.8	0.028	0.7 ± 1.3	0.3 ± 0.6	0.027
Clinical pregnancy rate (%)/per cycle	18.6	14.3	0.453	16.1	8.2	0.179
Cumulative pregnancy rate (%)	28.6	18.0	0.166	31.2	10.6	0.022
Live birth rate (%)/per cycle	13.4	11.7	0.735	9.7	6.6	0.527
Cumulative live birth rate (%)	20.6	14.8	0.391	18.8	8.5	0.179

*Data are presented as mean ± standard deviation or %. FSH, follicle stimulation hormone*.

No major adverse effects were reported during the study period. However, some participants suffered from acne (3.1%), deepening of the voice (1.9%) or hair loss (1.3%), but all the adverse effects were minimal. No participants in the DHEA group withdrew due to adverse effects.

## Discussion

This retrospective cohort study is the first to assess the effects of DHEA on IVF outcomes in patients who fulfilled the POSEIDON group 4 criteria. In this study, the incidence of patients in POSEIDON group 4 was 17.7% (384/2,165), which was higher than the incidence of Bologna POR (19). This study demonstrated that DHEA supplementation was associated with higher follicular oocyte index, higher numbers of retrieved oocytes, metaphase II oocytes, fertilized oocytes, day 3 embryos and top-quality day 3 embryos. Although the clinical pregnancy rate, live birth rate, and cumulative live birth rate were similar between the two groups, a higher cumulative pregnancy rate and lower cancellation rate were observed in the DHEA group than in the non-DHEA group. Moreover, the multivariate analysis revealed a 3.37-fold increase in the possibility of retrieving more than 3 oocytes (95% CI 1.64–6.96, *p* = 0.001) in the POSEIDON group 4 patients with DHEA supplementation compared to those without DHEA supplementation. Numerous studies have shown that DHEA supplementation seems to improve the IVF outcomes of patients with poor ovarian response, patients with diminished ovarian reserve, and patients of advanced age (5–7, 20). A prospective study included 25 patients with diminished ovarian reserve, showing significant increases in the numbers of retrieved oocytes, fertilized oocytes, day 3 embryos and average embryo scores per oocyte after DHEA treatment (8). A randomized controlled trial enrolled PORs according to the Bologna criteria, suggesting that DHEA supplementation resulted in higher numbers of oocytes, metaphase II oocytes, fertilized oocytes, and grade I embryos and a higher ongoing pregnancy rate and live birth rate (5). The meta-analyses also revealed that DHEA pretreatment may be associated with an improved pregnancy rate or live birth rate for PORs or patients with diminished ovarian reserve (10, 11). However, additional larger, multicenter, and randomized trials are needed to confirm the beneficial effects of DHEA on reproductive outcomes.

The actions of DHEA occur mainly via its conversion to the androgen, which acts locally to regulate follicular and luteal function through an autocrine and/or paracrine effect. Androgen receptors are expressed in granulosa cells, theca cells and oocytes in most follicular stages, especially in preantral and growing antral follicles. Androgen receptors decrease as follicles grow to the preovulatory stage (21, 22). Indeed, androgens have been reported to be involved in the initiation of primordial follicle recruitment, in the stimulation of the early stages of follicular development by increasing FSH receptor expression and in increasing the number of growing follicles by reducing follicular atresia (23–26). However, androgenic activity secondary to DHEA or testosterone supplementation is different. Testosterone directly interacts with the androgen receptor and exerts maximal effects, while DHEA displays multiple cell-specific controls that adjust androgen formation and inactivation to its local needs (27). Furthermore, active testosterone appears in the blood before exposure to its target tissues, while DHEA is converted to androgen intracellularly without significant release of active testosterone into the blood (27). Therefore, DHEA supplementation seems to result in more precise actions and cause fewer adverse effects than testosterone supplementation in clinical practice.

The detailed molecular mechanisms of the effect of DHEA on oocytes, cumulus cells and granulosa cells are still unclear. Our previous studies showed that DHEA might be associated with improvements in mitochondrial function and the regulation of mitochondrial dynamics and homeostasis (28–30). DHEA supplementation could increase mitochondrial mass and mitochondrial dehydrogenase activity, reduce mitochondrial reactive oxidative species generation and diminish the loss of mitochondrial membrane potential in human cumulus cells or human HO23 granulosa cells (28, 29). Additionally, DHEA treatment restores mitochondrial morphology, enhances mitochondrial fusion and reduces mitochondrial fission and mitophagy in human cumulus cells (30). However, further studies are required to explore the molecular mechanisms underlying DHEA actions.

The retrospective design and relatively small sample size of this study presented major limitations. In addition, the lack of a power calculation was considered when examining the lack of significant differences in this study. Furthermore, different gonadotropins and methods of oocyte trigger were used in this study. Although the percentage of using different gonadotropins and oocyte trigger methods were similar between groups, these may lead to possible bias. Another possible bias may originate from different time from trigger to ovum pickup even though there was no difference in terms of mean time between groups. Besides, a total of 209 patients with 297 cycles were included in this study, implying that some patients might be included more than one time. The strength of this study was that all the IVF cycles were based on the same protocol and performed by the same physician and embryologist, which may minimize bias. Poor prognosis patients were classified by POSEIDON groups. The same DHEA dose was used in all treated patients.

In conclusion, DHEA pretreatment prior to an IVF cycle may improve the number of oocytes and embryos in patients who fulfill the POSEIDON group 4 criteria, regardless of whether the patients are aged ≤ 40 or > 40 years. Moreover, DHEA supplementation may raise cumulative pregnancy rate, especially in patients aged older than 40. However, larger well-designed studies are needed to further demonstrate the clinical relevance of DHEA on improving reproductive outcomes.

## Data Availability Statement

The datasets used and analyzed during the current study are available from the corresponding author on reasonable request.

## Ethics Statement

The study conformed to the Declaration of Helsinki for Medical Research involving Human Subjects. Additionally, approval was obtained from the institutional review board at Kaohsiung Veterans General Hospital, with the identifier VGHKS19-CT3-01. The study was performed in accordance with approved guidelines.

## Author Contributions

P-HW and Z-HW contributed conception and design of the study. C-UC organized the database. L-TL performed the statistical analysis. S-NC wrote the first draft of the manuscript. L-TL and K-HT wrote sections of the manuscript. All authors contributed to manuscript revision, read, and approved the submitted version.

### Conflict of Interest

The authors declare that the research was conducted in the absence of any commercial or financial relationships that could be construed as a potential conflict of interest.

## Abbreviations

AFC: antral follicle count
AMH: anti-Müllerian hormone
CI: confidence interval
DHEA: dehydroepiandrosterone
FSH: follicle stimulation hormone
GnRH: gonadotropin-releasing hormone
hCG: human chorionic gonadotropin
IVF: *in vitro* fertilization
OR: odd ratio
POR: poor ovarian responder
POSEIDON: Patient-Oriented Strategies Encompassing IndividualizeD Oocyte Number.
